# Setting sub-organellar sights: accurate targeting of multi-transmembrane-domain proteins to specific chloroplast membranes

**DOI:** 10.1093/jxb/erx351

**Published:** 2017-11-01

**Authors:** Vivien Rolland, Benjamin D Rae, Benedict M Long

**Affiliations:** 1 CSIRO Agriculture and Food, Acton ACT, Australia; 2 ARC Centre of Excellence for Translational Photosynthesis, Division of Plant Science, Research School of Biology, The Australian National University, Acton ACT, Australia

**Keywords:** Chloroplast membranes, transmembrane proteins, protein targeting, SCY components, SEC translocase

## Abstract

This article comments on:

**Singhal R, Fernandez DE.** 2017. Sorting of SEC translocase SCY components to different membranes in chloroplasts. Journal of Experimental Botany **68,** 5029–5043.

**Engineering novel chloroplast functions requires an understanding of how to accurately target proteins to specific chloroplast sub-compartments. This is particularly difficult in the case of membrane proteins where localization can be confounded by multiple membrane types. In an elegant study, Singhal and Fernandez (2017) have now provided greater insight into this challenge by dissecting out the signals that control differential targeting of two related proteins to specific chloroplast membranes. Further development of this information should inform attempts to direct engineered proteins to specific sub-organellar membranes, bringing about desired phenotypic changes.**

Plant function relies on correct expression and localization of proteins to specific tissues, cells, and organelles and their sub-compartments. In the case of chloroplasts, these sub-compartments include the outer- and inner-envelope membranes, the thylakoid membranes, the inter-membrane space, the stroma, and the thylakoid lumen. Sub-organellar targeting is complicated by the fact that approximately 95% of chloroplastic proteins (~3000 proteins) are encoded in the nuclear genome, so must be imported into the organelle and subsequently targeted to the correct sub-compartment (Sugiura, 1989; Martin *et al.*, 2002; Timmis *et al.*, 2004). While proteins can enter chloroplasts via several pathways, most rely on an N-terminal transit peptide which directs them to the surface of the chloroplast where they can interact with the TIC/TOC protein import machinery (Jarvis, 2008; Li and Chiu, 2010). Considerable effort has led to a growing understanding of the make-up and regulation of the TIC/TOC apparatus, and how soluble proteins utilize this machinery to enter chloroplasts (Jarvis, 2008; Li and Chiu, 2010; Sjuts *et al.*, 2017). This knowledge has, for instance, enabled the design of strategies to target non-chloroplastic proteins to the stroma (Comai *et al.*, 1988; Bionda *et al.*, 2010; Rolland *et al.*, 2016).

Several hundred nuclear-encoded proteins localize to the internal membranes of the chloroplast, the inner-envelope and thylakoid membranes (Ferro *et al.*, 2010; Simm *et al.*, 2013; Gutierrez-Carbonell *et al.*, 2014). In the inner-envelope membrane these proteins regulate functions such as import and export, while in the thylakoid membrane they are involved in processes such as photosynthesis (Ferro *et al.*, 2010; Simm *et al.*, 2013; Gutierrez-Carbonell *et al.*, 2014). For these functions to be achieved and maintained, a specific set of transmembrane proteins must be accurately targeted to either of these membranes. Despite playing key roles in chloroplasts, few studies have concentrated on how transmembrane proteins reach their final sub-compartment, and in most cases the focus has been on proteins with a single hydrophobic domain (e.g. Li and Schnell, 2006; Tripp *et al.*, 2007; Firlej-Kwoka *et al.*, 2008; Viana *et al.*, 2010; Froehlich and Keegstra, 2011; Oh and Hwang, 2015). This focus has resulted in scant understanding of the protein motifs responsible for differential targeting of proteins with multiple transmembrane domains. It is therefore significant that Singhal and Fernandez (2017) have deciphered the sequences involved in targeting two related large proteins with multiple transmembrane domains to either of the two internal membranes of chloroplasts. Here, we place this study in the context of previous knowledge to present new avenues for accurate targeting of foreign proteins to specific chloroplast membranes, allowing the implementation of novel functions to improve plant performance.

## Differential targeting of chloroplastic proteins with a single transmembrane domain

The first significant step in understanding how transmembrane proteins are differentially sorted to internal chloroplastic membranes came from work by Froehlich and Keegstra (2011). Their research used nuclear-encoded proteins, possessing a single transmembrane domain and localizing either in the inner-envelope membrane or the thylakoids, to investigate the role of their hydrophobic membrane-spanning domain in differential targeting. Interestingly, when the transmembrane domain of the inner-envelope membrane protein Arc6 was swapped for that of a thylakoid-localized protein (STN8 or Plsp1), Arc6 localized in the thylakoid membrane; the converse occurred when the transmembrane domain of STN8 or Plsp1 was replaced with that of Arc6 (Box 1A; Froehlich and Keegstra, 2011). Their take-home message was that once in the chloroplast, the single transmembrane domain of these proteins is sufficient to determine the membrane in which they localize. While this provides insight into the role of transmembrane domains in targeting to specific chloroplast membranes, the underpinning mechanism remained unidentified.

**Box 1. Chloroplast inner-envelope and thylakoid membrane targeting signals**

Limited research investigating the targeting of membrane proteins to either the inner-envelope (IEM) or thylakoid (THY) membranes of plant chloroplasts has highlighted increasing detail about the importance of N-terminal peptide and transmembrane domain (TMD) sequences in leading proteins to their destination (A–C). The work of Singhal and Fernandez (C) provides an advance which suggests new routes to manoeuvre complex, multiple transmembrane domain cargoes to either internal membranes of chloroplasts with greater accuracy (D). Prot A: Protein A.

**Figure:**
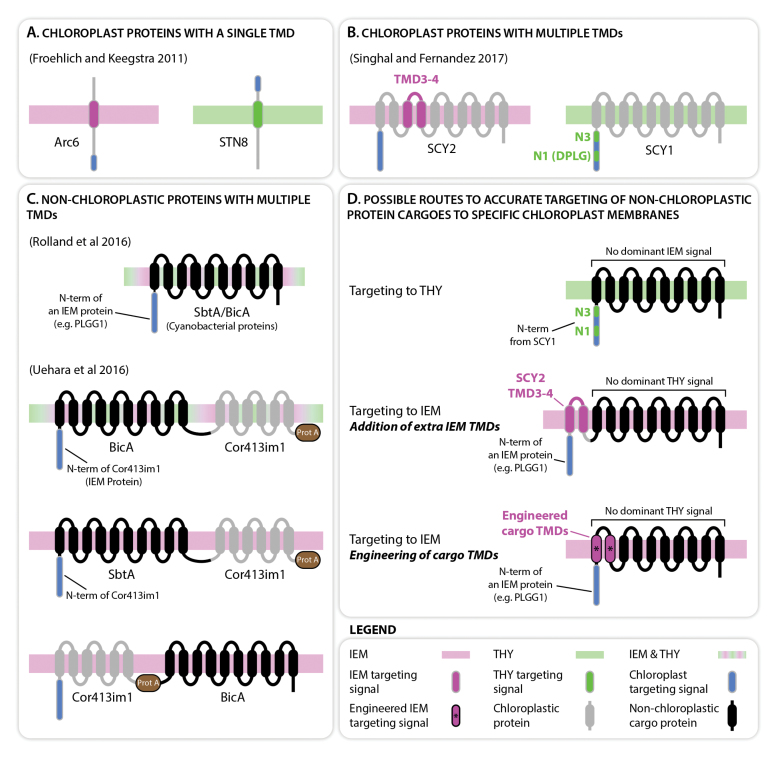

## Differential targeting of chloroplastic multi-transmembrane-domain proteins

The selective localization of proteins with multiple transmembrane domains poses a more complex set of questions. In these cases, the existence of more than one hydrophobic domain appears to lend itself to a greater chance of mistargeting to any of the membranes it encounters, from translation to its intended destination within the cell. Few studies have investigated the role played by individual transmembrane domains of multi-transmembrane-domain proteins in targeting to specific chloroplast membranes. One such example is a recent study by Okawa *et al.* (2014), in which the authors created a series of deletions in the nuclear-encoded, inner-envelope membrane protein Cor413im1 (six transmembrane domains) and assessed the localization of the resulting proteins. The main finding of this investigation was that the fifth transmembrane domain of Cor413im1 plays a role in targeting of the protein to the inner-envelope membrane. However, this domain alone was not sufficient for targeting to the correct chloroplast membrane, and instead the resulting protein localized to the thylakoids and stroma (Okawa *et al.*, 2014). This somewhat confusing result suggests that accurate targeting of Cor413im1 relies on a combination of signals, and these have yet to be identified.

The question as to what signals control the differential sorting of proteins to either of the two internal chloroplast membranes remained unanswered. Singhal and Fernandez (2017) designed a sophisticated study to investigate this problem, using two related nuclear-encoded Arabidopsis proteins: SCY1, which localizes in the thylakoids; and SCY2, which is targeted to the inner-envelope membrane (Box 1B). Both proteins are similar in structure, with 10 transmembrane domains each. This enabled the construction of a series of SCY1/SCY2 hybrids, where specific domains were swapped without disrupting protein topology. Protoplasts were transformed with these hybrids, and protein localization was analyzed by confocal microscopy. In the case of SCY2, the authors found that the fragment of SCY2 spanning transmembrane domains 3 to 4 was critical for its targeting to the inner-envelope membrane (Box 1B). One explanation for this could be that as they emerge from the TIC channel, these domains anchor the protein to the inner-envelope membrane. In the case of SCY1, the authors showed that its soluble N-terminus was essential for its localization in the thylakoids (Box 1B). They could further dissect this domain and showed that fragments called N1 and N3 were involved in targeting to thylakoids. Interestingly, N1 contains a conserved DPLG amino acid motif which is absent in SCY2. This motif is found in other thylakoid proteins and was previously shown to interact with the stromal chaperone SRP43, which promotes integration in the thylakoid membrane (Tu *et al.*, 2000; Stengel *et al.*, 2008). Singhal and Fernandez propose that targeting of SCY1 is mediated by SRP43.

## Targeting non-chloroplastic proteins with multiple transmembrane domains to chloroplast membranes

The work of Singhal and Fernandez (2017) elucidates the potential to specifically target complex, multi-transmembrane-domain proteins to different membranes within chloroplasts. This task is imperative in current plant engineering projects where the localization of specific membrane functionality is fundamental to successful outcomes. An example of this type of task is the generation of a cyanobacterial CO_2_-concentrating mechanism in C_3_ plant chloroplasts (Long *et al.*, 2016). In such a system, the generation of a high chloroplastic bicarbonate concentration is central and relies on the correct location and orientation of multi-transmembrane-domain bicarbonate pumps and ion transporter proteins (Rae *et al.*, 2017). Two recent examples highlight the difficulty of directing the cyanobacterial bicarbonate transporters BicA and SbtA, expressed in the plant nuclear genome, to the chloroplast inner-envelope membrane (Rolland *et al.*, 2016; Uehara *et al.*, 2016). BicA and SbtA are large proteins with 14 and 10 hydrophobic domains, respectively (Price and Howitt, 2014). In an attempt in our laboratory, we fused the N-terminus (~90–115 amino acids, no transmembrane domain) of several nuclear-encoded, inner-envelope-localized Arabidopsis proteins, to both cyanobacterial proteins (Rolland *et al.*, 2016). The N-terminus of PLGG1 proved to be the most potent leader, and targeted BicA or SbtA to chloroplasts. However, the chimeric proteins were found both in the inner-envelope membrane and in the thylakoids, indicating that while the plant leader sequence was sufficient for import, once inside the chloroplast the cyanobacterial sequence contributed to dual targeting (Box 1C). Interestingly, when BicA was expressed from the chloroplast genome it also localized to both membranes (Pengelly *et al.*, 2014). In Uehara *et al.* (2016) the authors targeted BicA and SbtA using the N-terminus of Cor413im1 as well as all, or most, of its transmembrane domains. The addition of Cor413im1 transmembrane domains enabled specific targeting of BicA and SbtA to the inner-envelope membrane. While specific targeting could be achieved by placing SbtA in front of Cor413im1 transmembrane domains, BicA had to be placed after Cor413im1 to prevent dual targeting to thylakoids (Uehara *et al.*, 2016). This is possibly because the inner-envelope transmembrane domains, which in this construct were first to emerge from the TIC channel, helped anchor BicA in the inner-envelope membrane. These studies highlight the ability to target complex transmembrane proteins to chloroplast membranes, but their function as transporters in this organelle remains to be tested.

## Possible ways to target cargo proteins to specific chloroplast membranes

In light of the work of Singhal and Fernandez (2017), and leveraging from previous knowledge (e.g. Froehlich and Keegstra, 2011; Rolland *et al.*, 2016; Uehara *et al.*, 2016), several approaches to target non-chloroplastic proteins to specific chloroplast membranes now arise.

To target foreign cargoes to thylakoid membranes, the most tempting approach is to fuse the target protein to the N-terminal signals present in the protein sequence of SCY1 (Box 1D). These signals are likely to be sufficient to guide the chimeric protein to chloroplasts, where they may be able to traffic the protein to the thylakoid membrane. This approach would need to ensure that the multiple transmembrane domain component of the protein cargo does not contain a dominant signal that could confuse directionality and lead to mistargeting of some, or all, of the protein to the inner-envelope membrane.

To target foreign cargoes to the inner-envelope membrane, the outcomes of the work by Singhal and Fernandez indicate two potential routes to successful localization (Box 1D). Initially, both routes require successful targeting to chloroplasts, which could be achieved by fusing the N-terminus of an inner-membrane protein to the cargo (Rolland *et al.*, 2016). After this point, one strategy could include the fragment of SCY2 spanning transmembrane domains 3 to 4 to anchor the chimera to the inner-envelope membrane. Alternatively, a second approach would be to re-engineer the initial transmembrane domains of the cargo protein to ensure that they resemble those of inner-membrane proteins (Froehlich and Keegstra, 2011; Singhal and Fernandez, 2017). With either approach, it is essential to ensure that no dominant thylakoid signal is present within the cargo protein sequence, thus decreasing the potential for mistargeting that has been observed in more recent engineering attempts (Pengelly *et al.*, 2014; Rolland *et al.*, 2016).

Complex chloroplast engineering projects demand accurate membrane localization for functionality. Singhal and Fernandez provide new insights into targeting large, multi-transmembrane-domain proteins to specific chloroplast membranes, providing a solid framework from which researchers can formulate gene construct designs for successful outcomes in functional plant engineering. The challenge now is to implement accurate protein targeting strategies informed by this work, while maintaining protein function.
